# Uncertainty quantification in multi-parametric MRI-based meningioma radiotherapy target segmentation

**DOI:** 10.3389/fonc.2025.1474590

**Published:** 2025-01-28

**Authors:** Lana Wang, Zhenyu Yang, Dominic LaBella, Zachary Reitman, John Ginn, Jingtong Zhao, Justus Adamson, Kyle Lafata, Evan Calabrese, John Kirkpatrick, Chunhao Wang

**Affiliations:** ^1^ Department of Radiation Oncology, Duke University, Durham, NC, United States; ^2^ Medical Physics Graduate Program, Duke Kunshan University, Kunshan, Jiangsu, China; ^3^ Department of Radiology, Duke University, Durham, NC, United States; ^4^ Department of Electrical Engineering, Duke University, Durham, NC, United States

**Keywords:** meningioma, uncertainty quantification, radiation therapy, deep learning, auto-segmentation

## Abstract

**Purpose:**

This work investigates the use of a spherical projection-based U-Net (SPU-Net) segmentation model to improve meningioma segmentation performance and allow for uncertainty quantification.

**Methods:**

A total of 76 supratentorial meningioma patients treated with radiotherapy were studied. Gross tumor volumes (GTVs) were contoured by a single experienced radiation oncologist on high-resolution contrast-enhanced T1 MRI scans (T1ce), and both T1 and T1ce images were utilized for segmentation. SPU-Net, an adaptation of U-Net incorporating spherical image projection to map 2D images onto a spherical surface, was proposed. As an equivalence of a nonlinear image transform, projections enhance locoregional details while maintaining the global field of view. By employing multiple projection centers, SPU-Net generates various GTV segmentation predictions, the variance indicating the model’s uncertainty. This uncertainty is quantified on a pixel-wise basis using entropy calculations and aggregated through Otsu’s method for a final segmentation.

**Results/conclusion:**

The SPU-Net model poses an advantage over traditional U-Net models by providing a quantitative method of displaying segmentation uncertainty. Regarding segmentation performance, SPU-Net demonstrated comparable results to a traditional U-Net in sensitivity (0.758 vs. 0.746), Dice similarity coefficient (0.760 vs. 0.742), reduced mean Hausdorff distance (mHD) (0.612 cm vs 0.744 cm), and reduced 95% Hausdorff distance (HD95) (2.682 cm vs 2.912 cm). SPU-Net not only is comparable to U-Net in segmentation performance but also offers a significant advantage by providing uncertainty quantification. The added SPU-Net uncertainty mapping revealed low uncertainty in accurate segments (e.g., within GTV or healthy tissue) and higher uncertainty in problematic areas (e.g., GTV boundaries, dural tail), providing valuable insights for potential manual corrections. This advancement is particularly valuable given the complex extra-axial nature of meningiomas and involvement with dural tissue. The capability to quantify uncertainty makes SPU-Net a more advanced and informative tool for segmentation, without sacrificing performance.

## 1 Introduction

Meningiomas are the most common primary brain tumors; while usually benign, these tumors can lead to significant morbidity (1–3). Given their origin from meningothelial cells, meningiomas can have significant skull-brain interface characteristics with extracranial extension and spread along the dura (1, 2). Treatment is often individualized and planned based on anatomical location as well as World Health Organization (WHO) classification (1–3). Treatment typically involves a combination of observation, surgical resection, and radiation therapy. Radiation therapy plays a large role in mitigating recurrence after resection (4–8) for WHO grade 1 and grade 3 tumors as well as being the primary treatment modality for unresectable meningiomas (1, 9, 10). Auto-segmentation tools, i.e. deep neural network (DNN) models, have promising implications for use in radiation therapy planning which require a gross tumor volume (GTV) to be contoured (11–14). These DNNs have the potential to reduce contour variability, leading to improved clinical outcomes while increasing workflow efficiency (11–13). Most automated brain tumor segmentation efforts to date have been focused on gliomas (15–17), despite meningiomas being the most common type of primary brain tumor. The few segmentation studies on meningiomas are commonly related to general volume segmentation, for clinical use in analyzing tumor growth (18–21). These meningioma segmentation studies are based on contrast-enhancing tissue observed in magnetic resonance imaging (MRI) (18–21). Auto-segmentation of meningioma for radiotherapy targeting is even less studied. Meningioma patients that are treated with radiation are either post-resection or have unresectable tumors where delineation of meningioma GTVs incorporates an additional layer of complexity. GTV segmentation proves to be a challenge due to meningioma’s extra-axial characteristic locations and up to 72% having an associated dural tail (22). Meningioma GTV segmentation may include the dural enhancement and invaded bone in addition to the main contrast-enhancing mass (23, 24). The extension of the dural tail segmentation can be subjective and difficult to define.

High-performing segmentation DNNs can serve as a valuable tool; however, model performance is heavily reliant on the robustness of the training data. When used for complex structures and at higher risk clinical scenarios, the accuracy and accountability of these models are an area of concern (25). Predictions made without uncertainty quantification may not be trustworthy causing DNN models to create a false sense of certainty (25, 26). DNNs sometimes make unexpected incorrect predictions and the need for uncertainty quantification is apparent. There are numerous studies based on analyzing model uncertainty through two approaches: model-based uncertainty (epistemic) and data-based uncertainty (aleatoric) (25, 26). Epistemic uncertainty quantification methods include Bayesian neural networks (27, 28) and Monte Carlo dropout (29, 30), while aleatoric uncertainty methods include test-time augmentation (31, 32). However, there is not a standard method to quantify uncertainty. Specifically, a pixel-wise uncertainty quantification method remains unavailable. A pixel-wise uncertainty metric would quantify the likelihood that each pixel belongs in the segmentation region, providing a corresponding uncertainty quantification with each segmentation prediction (33).

This innovative work investigated the use of spherical image projection to quantify DNN segmentation aleatoric uncertainty for meningioma radiotherapy target delineation. Inspired by spherical camera image processing (34), our group developed a novel spherical projection-based method to enable image segmentation uncertainty estimation of DNN models. Such design emphasizes locoregional image texture details near the projection center while maintaining the global anatomy information (35). Previously, our group has demonstrated that our spherical projection design can successfully quantify uncertainty in multi-parametric MRI-based glioma segmentation (33). This work is the first of its kind in deep learning-based meningioma segmentation with uncertainty analysis. This feature serves as an invaluable tool for clinicians, offering visual cues that facilitate efficient correction of deep learning segmentation outputs. We hypothesize that the implementation of spherical projection-based segmentation and uncertainty quantification can hold great value in meningioma radiotherapy target delineation.

## 2 Materials and methods

### 2.1 Patient data

**Figure 1:**
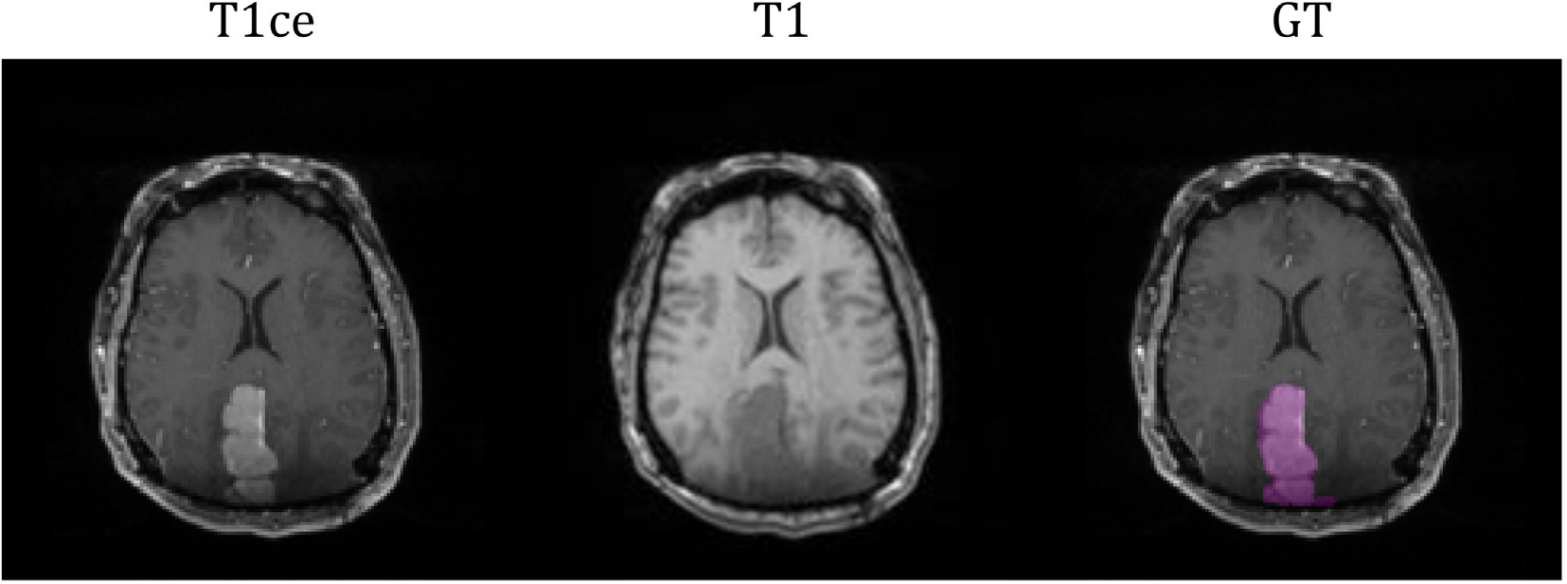
MRI sequences of T1ce and T1 with corresponding ground-truth segmentation.

The study cohort included 76 meningioma patients with intracranial meningiomas of varying WHO grades. All patients underwent radiation therapy within the Duke University Healthcare System. This study (Pro00110695) was conducted under the approval of Duke University Health System Institutional Review Board. Exclusion criteria included tumors with a volume less than 10 mm^3^, infratentorial located tumors, and postoperative cases with no residual gross tumor. Each patient had two standard MRI sequences as a multi-parametric MRI (mpMRI) protocol: T1-weighted (T1), and contrast-enhanced T1-weighted (T1ce). MRI images were pre-processed by co-registering to the patient’s planning computed tomography (CT) images and interpolated to a resolution of 1x1x1 mm^3^. The GTV for the purpose of radiation treatment was contoured by a radiation oncology physician, serving as the ground truth (GT) in this segmentation study. Out of the cohort, 6 patients’ GTVs were comprised with more than one lesion. The average GTV volume was 31.84 cc (20-80% percentile range: 3.77- 48.16 cc). **Figure 1** illustrates an example of the two MRI sequences with a corresponding ground-truth segmentation.

### 2.2 Model design

#### 2.2.1 Spherical projection

**Figure 2:**
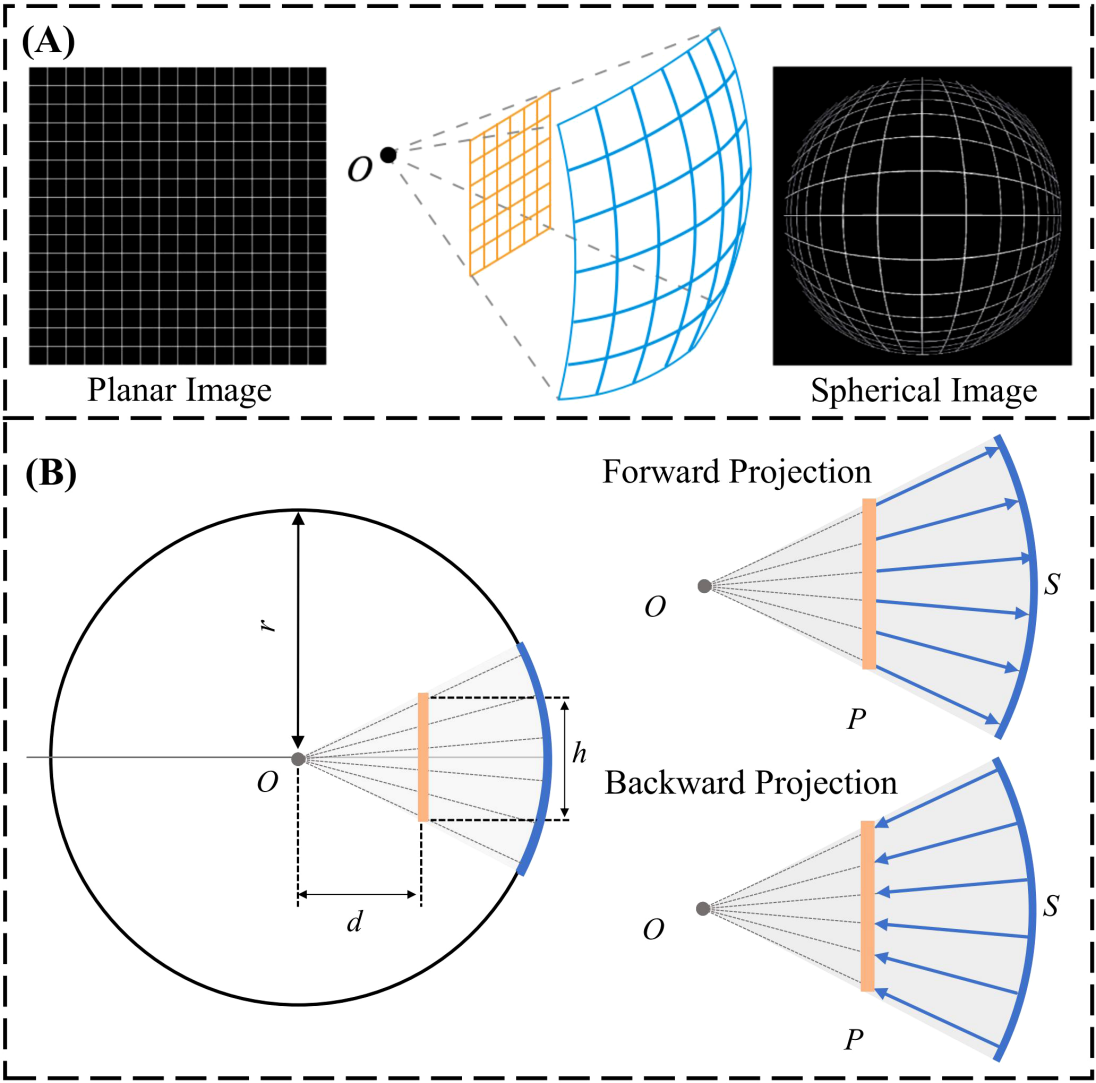
**(A)** General schematic of spherical image projection. **(B)** Forward projection: projection of a planar image onto a spherical surface. Backward projection: projection of a spherical image onto the Cartesian plane.

The spherical image projection methodology proposed by Yang et al. (33) was adopted in this work; where it was shown to increase segmentation accuracy and quantify segmentation uncertainty in the use of glioblastoma segmentation. Specifically, the planar MRI images are projected onto a sphere as a spherical MRI image. Therefore, each pixel within the original image can be projected onto the spherical surface as a form of non-linear image transformation. Spherical image projection causes inhomogeneous scaling over the original image, illustrated in **Figure 2A**. Image details near the center become magnified, enhancing local image details, while preserving the global field-of-view (FOV).

The forward projection is defined as the projection of the planar image to a spherical surface. Computationally, an arbitrary sphere is defined with a center *O* and a radius of *r*, illustrated laterally in **Figure 2B**. The planar image *P* is placed at a projection distance *d* from the center of the sphere. The planar image with a size of *h* x *h* is projected onto the spherical surface by quantifying *h*, *d* and *r* to determine the projection geometry. The spherically projected image is subsequently obtained by projecting each individual planar image pixel onto the pre-defined sphere along the radius. Backward projection is the inverse operation, projecting the spherical image back to planar, illustrated laterally in **Figure 2B**. The design of the sphere (*h*=0.5, *r*=1, *d*=0.3) from our group’s prior effort (33) with the smallest image quality degradation, quantified using the structural similarity index (SSIM), was adopted for this work.

**Figure 3:**
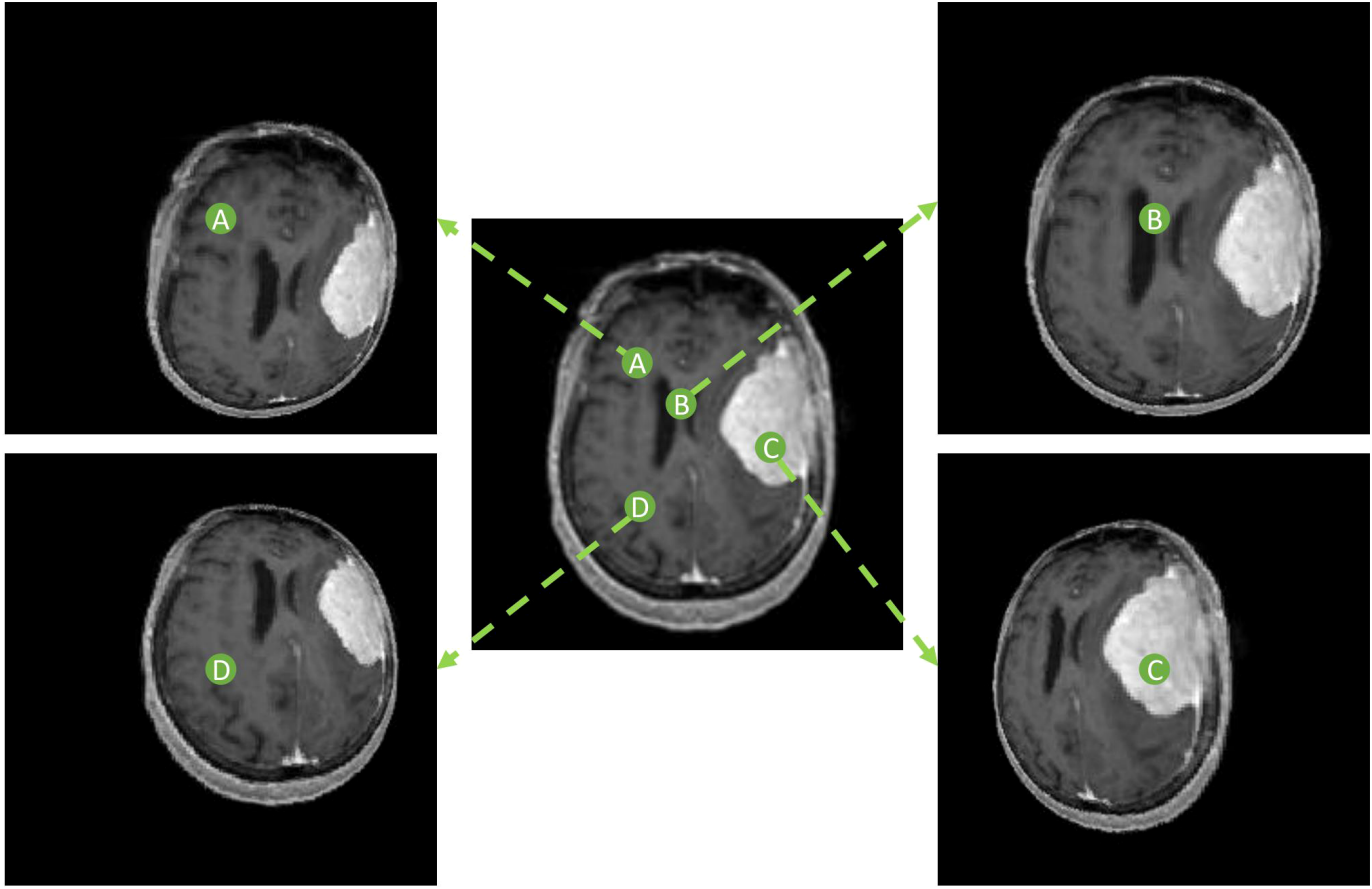
Spherically projected images with varying projection center locations. Four different projection centers **(A-D)** are shown with respective spherical projections.

The spherically projected images were processed as nonlinear image transforms and therefore, reside in the Cartesian plane. The locoregional magnification is specified by the location of the designated projection center *O*. A group of spherical projected images can be created by utilizing projection centers at varying locations in the original planar image, illustrated in **Figure 3**. The variation in segmentation predictions arising from multiple spherical projection images reflects the internal uncertainty of the deep learning model.

#### 2.2.2 Overall model design

**Figure 4:**
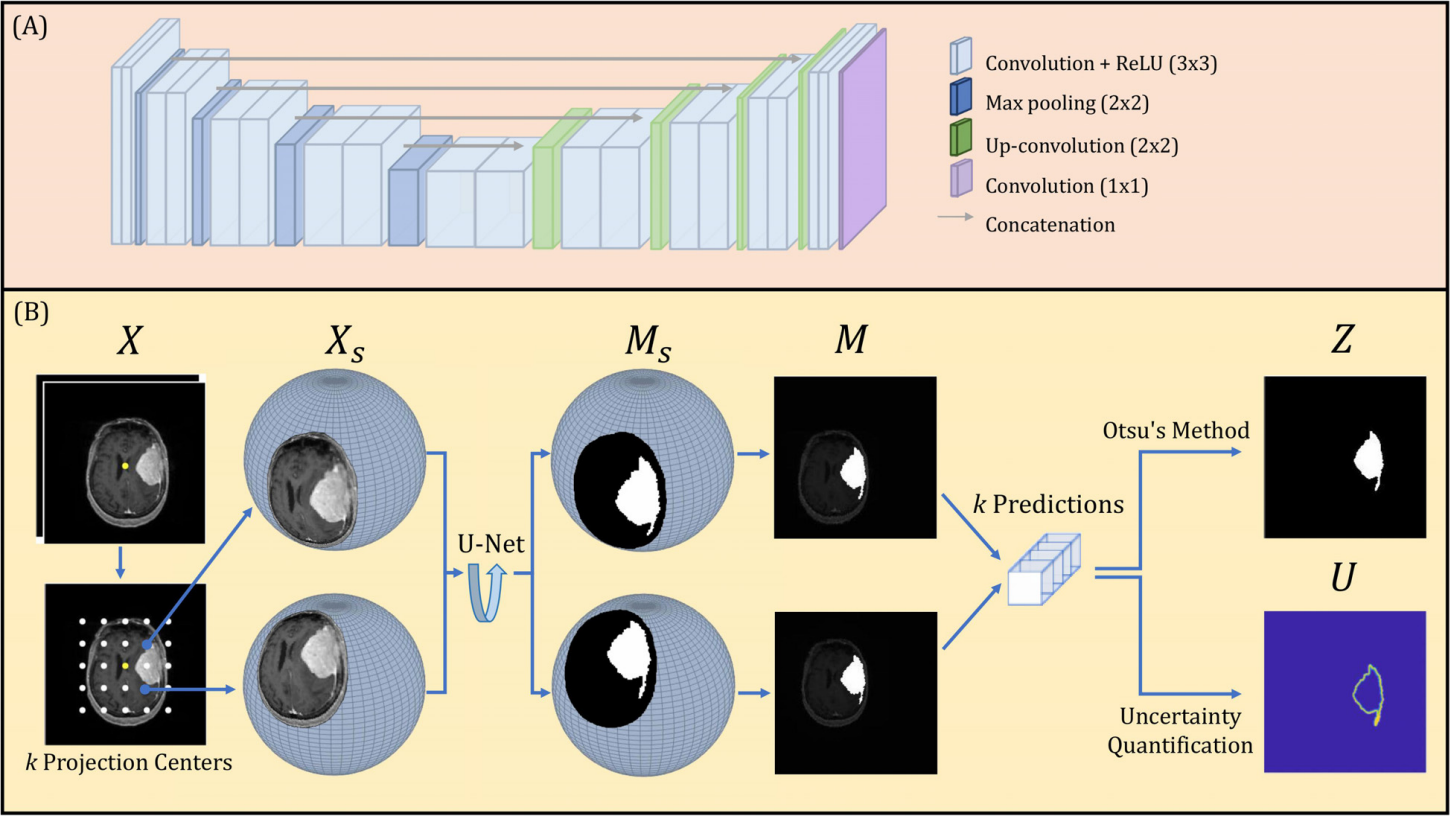
**(A)** U-Net architecture. **(B)** Schematic diagram of overall SPU-Net model workflow.

A U-Net deep learning network was constructed, composed of encoding and decoding segments, illustrated in **Figure 4A**. Each encoding layer is composed of two sequential convolutions with rectified linear Unit (ReLU) operations followed by a max pooling layer. Each decoding layer is constructed as a concatenation followed by two sequential up-convolutions with ReLU operations. The encoding and decoding layers are connected through concatenation operations. The final 1x1 convolution layer with a sigmoid operation produces the binary segmentation prediction.

The proposed spherical projection-based U-Net (SPU-Net) workflow followed our previous work (33), including both forward and backward spherical projection, which is illustrated in **Figure 4B**. The original planar 2-channel mpMRI input is denoted as *X*, with the image center shown as a yellow dot. While maintaining the pre-defined sphere geometry, multiple forward-projected spherical images, *Xs*, can be obtained using different projection centers. By varying the location of the projection center, the locoregional magnification is varied. These projection centers are uniformly distributed around the image center, with *k* projection centers, illustrated as white dots in **Figure 4B**. In this study, a total of *k*=121 projection centers were used. The SPU-Net workflow for a given 2-channel mpMRI image set *X* is summarized as:

1. Original input 2 channel mpMRI image set *X* with dimension of 192 x 192 x 2.
2. Forward projection with *k* different projection centers obtains a set of *k* 2-channel spherically projected images *Xs*. Performing spherical projection and use of padding scales the input tensor to *k* x 256 x 256 x 2.
3. The output of the U-net is the spherically projected segmentation masks, represented as a set of *k* probability distribution maps on the spherical plane *Ms*. These are obtained as the output tensor with dimension of *k* x 256 x 256 x 1.
4. Backward projection obtains a set of *k* probability distribution maps in the Cartesian plane *M.*
5. Binarization using Otsu’s method (36) provides the final segmentation result with a dimension of 256 x 256, rescaled to the original image size of 192x192.
6. Uncertainty quantification reflects the image segmentation uncertainty of each pixel with a final uncertainty map of dimension 256 x 256, rescaled to the original image size of 192x192.

The final segmentation result is obtained by binarization using Otsu’s method. Otsu’s method was applied to the sum of all model predictions for a given MRI slice to obtain a global threshold, *T*. The binarization operation analyzes each pixel (*i*,*j*) within *X* that is accompanied by *k* independent segmentation predictions within *M*. If the average value of a pixel (*i*,*j*) observed within *M* is above the threshold *T*, the pixel will be binarized as 1. The binarization operation to quantify the value of pixels (*i*,*j*) in *Z* is designed as:


$$\text{Z}\left(\text{i},\text{j}\right)=\{\begin{array}{ll} 1, & \;if\;\left(\sum_{\text{n}=1}^{v}{\text{M}}_{\left(\text{i},\text{j}\right)}^{\text{n}}\right)/\text{k }>T\\ 0, & \;otherwise \end{array}$$

Uncertainty *U* is quantified through the entropy of the *k* independent segmentation predictions. Given the probability distribution *M*, there are a total of *v* unique values, and the frequency observed of each unique value *v* is ${\hat{p}}_{\left(i,j\right)}^{v}$ . The uncertainty quantification for each pixel (*i*,*j*) in *U* is approximated as:


$$U_{\left(i,j\right)}=-\sum_{v=1}^{V}{\hat{p}}_{\left(i,j\right)}^{v}ln\left({\hat{p}}_{\left(i,j\right)}^{v}\right)\;$$

A final uncertainty score, adopted from the Brain Tumor Segmentation (BraTS) 2020 uncertainty challenge (QU-BraTS challenge 2020) (37) definition is quantified for use in future meningioma segmentation uncertainty studies. The uncertainty map, *U*, is normalized to 0-100, with uncertainty thresholds set as *τ = 1,2,3.,100. At each threshold, Z*(*i,j*) segmentation results with associated *U*(*i,j*) < *τ are considered in subsequent calculations. The filtered segmentation results in Z*(*i,j*) are compared with the corresponding ground truth using Dice similarity coefficient (DSC). At each *τ, the number of true positive and true negative pixels are obtained based on the Z*(*i,j*) after thresholding, specified as *TPτ and TNτ. The ratio of filtered true positive (FTP*) pixels and ratio of filtered true negative (*FTN*) pixels at each τ to the unfiltered *Z* (*TP_100_* and *TN_100_*) is defined as:


$$FTP_{\tau }=\;\frac{TP_{100}-TP_{t}}{TP_{100}}$$

$$FTN_{\tau }=\;\frac{TN_{100}-TN_{t}}{TN_{100}}$$

Based on these definitions, three curves and their corresponding area under the curve (AUC) can be obtained. *AUC_1_* is the area under the curve of the DSC over *τ, with a high AUC_1_* indicating accurate segmentation predictions have low uncertainty. Therefore, a high *AUC_1_* represents a high confidence in the correct segmentation results. *AUC_2_* is the area under the curve of *FTP* over *τ, such that (1-AUC_2_*) will penalize true positive predictions with associated low confidence. *AUC_3_* is the area under the curve of *FTN* over *τ, such that (1-AUC_3_*) will penalize true negative predictions with associated low confidence. Then, the final uncertainty score, *U-score*, is defined as:


$$Uscore=\;\frac{AUC_{1}+\left(1-AUC_{2}\right)+\left(1-AUC_{3}\right)}{3}$$

Collectively, the uncertainty score ranges from 0 to 1, which rewards high confidence in correct predictions and low confidence in incorrect predictions while penalizing low confidence for pixels with correct predictions. Therefore, the *U*-*score* is expected to be high when *Z*(*i,j*) is correct with a low *U*(*i,j*) and when *Z*(*i,j*) is incorrect with a high *U*(*i,j*). In other words, a high *U*-score is expected when (1) high confidence in the correct segmentation prediction and (2) low confidence in the incorrect predictions.

#### 2.2.3 Comparison study

The proposed spherical projection-based model, SPU-Net, was compared to a more traditional U-Net model. The U-Net model contained the same U-Net architecture backbone as SPU-Net, with only the pre and post processing altered (i.e. no spherical projection). This U-Net model utilized the same mpMRI image sets with test-time augmentation of 8 augmentations per MRI slice which includes rotations, translation, and flipping. In the comparative analysis, the SPU-Net model and the classic U-Net model were trained in the same fashion using a 7:3 train/test sample ratio with the same train/test samples. Randomization into training and testing groups was performed per subject with all meningioma tumor-bearing slices for each subject used for either training or testing. The number of image slices used for training, validation, and testing were 1762, 489, and 284. Both models were optimized for their specific data input. During training, the loss function was set as binary cross-entropy.

This comparison analysis was to evaluate the performance of the SPU-Net methodology to a baseline traditional segmentation model. The SPU-Net’s output combo of mask and uncertainty map was visually inspected against the U-Net’s output to examine the value for meningioma GTV segmentation purposes. The two models were compared quantitatively through metrics of accuracy, sensitivity, specificity, 2D Dice similarity coefficient, mean Hausdorff distance (mHD), and 95% Hausdorff distance (HD95). The mHD is the mean distance between the patient’s *GT* and *Z* surface points, while the HD95 is the 95^th^ percentile distance (38).

## 3 Results

**Figure 5:**
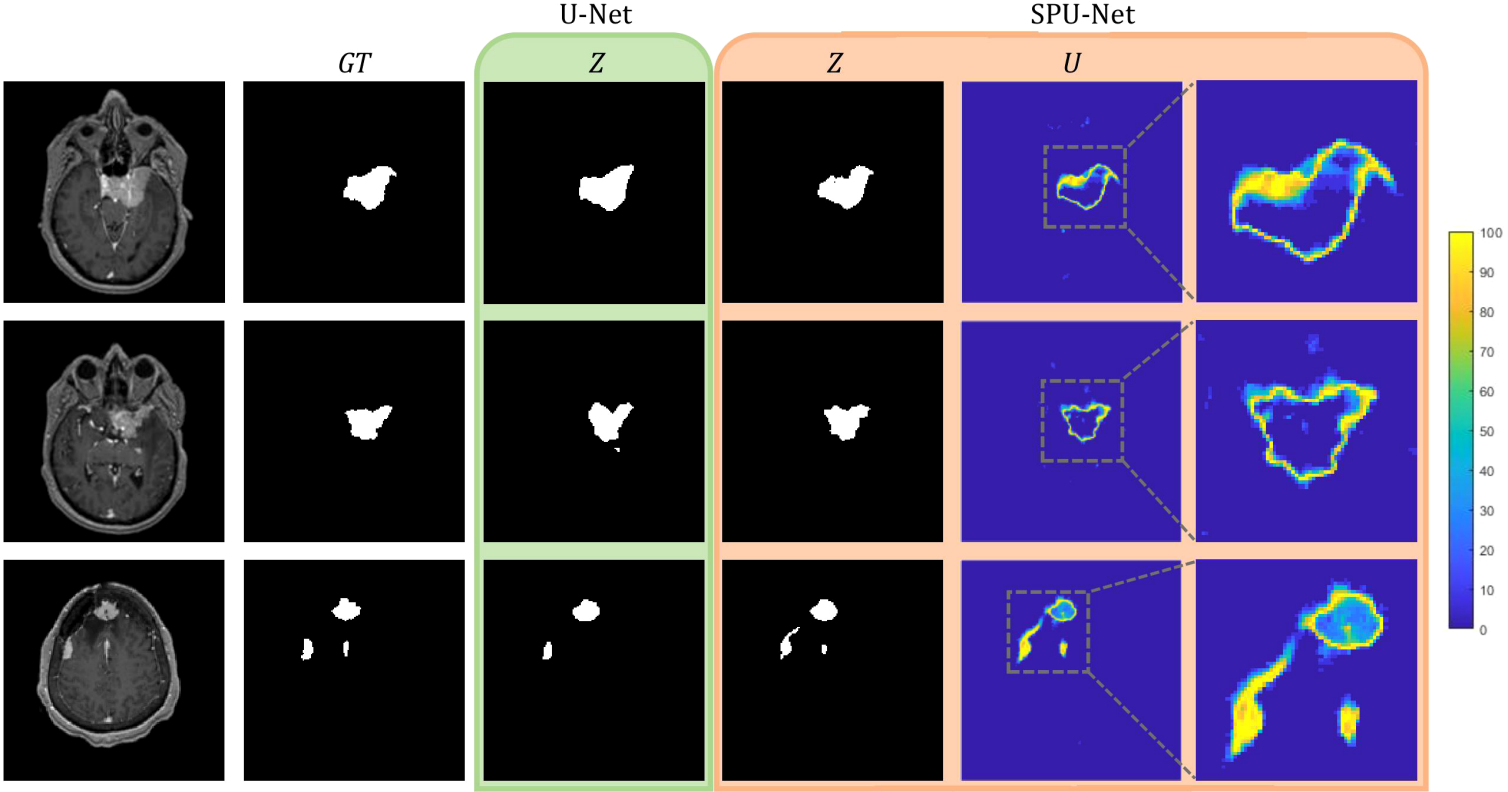
U-Net and SPU-Net output for three examples of MRIs and GTs.U-Net output of segmentation mask, Z. SPU-Net output of segmentation mask, Z and uncertainty map, U.

**Figure 5** shows three examples of outputs from the U-Net and SPU-Net models. In all three examples, SPU-Net’s segmentation displays more visual similarity to the GT compared to U-Net’s segmentation. The most obvious visual discrepancy is observed around the tumor edges and near dural vessels, for SPU-Net and more so for U-Net predictions. In the bottom examples, with 3 tumors, SPU-Net over-segmented the dural tail region of the left-most lesion with U-Net not able to segment the middle lesion. Similarly observed in other test cases, U-Net was observed to have difficulty delineating small lesions, especially those in proximity with vasculature. Generally, SPU-Net is observed to have better segmentations when meningiomas are near dural vessels and generally have better dural tail segmentations. SPU-Net can better delineate and differentiate between meningioma tissue and neighbouring blood vessels.

The SPU-Net’s segmentation mask and uncertainty map showcase a correlation. Segmentation errors are reflected in the uncertainty map with the region having high uncertainty. Accurate regions in the segmentation mask are reflected to yield low uncertainty. This observation meets the expectation of a favored *U* visualization. SPU-Net achieved a *U-score* of 0.718 ± 0.183, which will be used as benchmark results for future meningioma segmentation uncertainty studies.

**Table 1** summarizes the segmentation results evaluated between the SPU-Net and traditional U-Net models. While both models achieved acceptable results, SPU-Net achieved slightly higher sensitivity and DSC values. SPU-Net achieved comparable sensitivity (0.758 vs. 0.746) and Dice similarity coefficient (0.760 vs. 0.742) results from U-Net. SPU-Net also had smaller mHD (0.612 cm vs 0.744 cm) and smaller HD95 (2.682 cm vs 2.912 cm). Additionally, smaller variances are observed in SPU-Net results. These numerical result improvements, though promising, did not achieve statistical significance in Wilcoxon signed-rank tests.

**Table 1:** SPU-Net and U-Net accuracy, sensitivity, specificity, Dice coefficient, mean Hausdorff distance, and 95% Hausdorff distance results.

	Accuracy	Sensitivity	Specificity	DSC	mHD	HD95
U-Net	0.997 ± 0.002	0.746 ± 0.237	0.999 ± 0.001	0.742 ± 0.207	0.744 ± 1.724	2.912 ± 3.323
SPU-Net	0.997 ± 0.002	0.758 ± 0.182	0.999 ± 0.001	0.760 ± 0.143	0.612 ± 0.566	2.682 ± 2.435

## 4 Discussion

In this work, a novel method to quantify deep learning segmentation uncertainty was investigated for use in meningioma radiotherapy target delineation. This novel method of spherical projection allows for projecting planar MR images onto a spherical surface, which could be considered as a form of nonlinear image transformation. Utilizing multiple projection centers across the FOV, structures are magnified at varying scales. The spherical projection-based method obtains multiple independent segmentation predictions from a single mpMRI input and allows for improved segmentation and most importantly, uncertainty quantification.

Meningioma segmentation presents a significant challenge even for experienced radiation oncologists due to the inherent subjectivity surrounding the delineation of GTV boundaries. GTV boundaries often overlap with blood vessels specifically in the dural tail segmentation region, where the extent of the dural tail segmentation is based on judgement and experience. Some physicians may be more cautious and extend the GTV edges and dural tails further than others. SPU-Net addresses the challenge of meningioma segmentation and the major concern regarding DNN models of not having a confidence metric. SPU-Net provides a pixel-wise uncertainty map to accompany the binary segmentation prediction, to provide indication of prediction confidence. The pixel-wise uncertainty map in the same FOV as the segmentation mask substantially enhances the interpretability of a binarized segmentation mask. **Figure 5** illustrates the value of the uncertainty map. With meningioma being a more challenging segmentation task due to interobserver variability, there is a higher likelihood of segmentation errors where the uncertainty map offers visual cues on the anatomical locations of these errors. The uncertainty map highlights the areas of high uncertainty, predominantly observed around the GTV periphery, regions overlapping with dural vessels, and the dural tail region. This observation corresponds to known areas of clinical uncertainty for oncologists. This alignment between SPU-Net’s uncertainty map and the clinical challenges faced by radiation oncologists demonstrates its potential for clinical adoption, providing more interpretable and clinically relevant results that reflect oncologists’ real-world experiences.

The results indicate comparable performance between SPU-Net and U-Net in quantitative segmentation metrics of sensitivity, DSC, mHD, and HD95. However, SPU-Net was observed to handle complex meningioma shapes and dural tail segmentations more effectively compared to U-Net. By incorporating spherical magnification, the SPU-Net GTV segmentations had better dural tail segmentations compared to the traditional U-Net model (**Figure 5**). U-Net sometimes struggled to segment areas where the meningioma overlapped with dural vessels. In certain instances, U-Net failed to generate a prediction altogether in such complex regions, highlighting the limitations of the traditional model in handling these challenging anatomical features. SPU-Net’s ability to handle the challenging dural tail region more effectively highlights its potential. Given the extra-axial nature of meningiomas, segmentation difficulties were expected for both models, but any improvement, especially in the dural tail region, is notable.

This study highlights the importance of incorporating uncertainty quantification into deep learning models, specifically through the use of spherical projection-based DNNs for meningioma segmentation, a method that has also shown promise in glioblastoma segmentation. Though the results are not statistically significant, the technical innovation justifies further exploration. A key advantage of spherical projection is its ability to generate an uncertainty map that is spatially aligned with the segmentation prediction. This uncertainty map provides valuable insights into the underlying mechanisms driving the DNN’s decisions, enhancing interpretability. For clinical applications, the uncertainty map offers clinicians a clearer understanding of the model’s predictions, helping them make more informed decisions and increasing their confidence in adopting the model. Future studies with larger cohorts and in different clinical applications could provide a deeper understanding of SPU-Net’s full potential and its broader applicability across different tumor types and segmentation challenges. The use of spherical projection is a model-agnostic method, it can be applied to other DNN architectures and for other segmentation applications (35).

This study can be further analyzed and improved by being more inclusive to inter-personal variations in GTV segmentation and incorporating these into the study on a quantitative level. This study utilized a GTV contoured by one singular physician, which introduces bias to the study. A more accurate representation of the ground truth may be an average of multiple physicians’ GTV contours. The variability between multiple physicians’ GTV contours could also represent a clinically acceptable uncertainty range.

The challenge of meningioma GTV subjectivity in segmentation can also be addressed by incorporating a larger dataset; to support a publicly available large dataset, the image data included in this work will be submitted to the 2024 BraTS meningioma radiotherapy segmentation challenge. The results in this study showed promising improvements through the incorporation of spherical projections. This issue could be potentially overcome by utilizing a larger cohort and increasing the sample size. With a larger cohort, the study can also incorporate infratentorial located tumors. Infratentorial tumors are particularly challenging to segment due to the complex anatomy of the posterior fossa (a vast number of blood vessels) and frequent imaging artifacts observed in this region, making it particularly tricky for an MRI only based segmentation task. The inclusion of CT scans is being considered to facilitate infratentorial tumor segmentation. Expansion upon this current study will require re-optimizing the SPU-Net workflow to be more robust to the variation in anatomically located meningiomas.

## 5 Conclusion

A spherical projection-based U-Net (SPU-Net) was successfully developed for meningioma segmentation using multi-parametric MRI. In a comparison study against a classic U-Net, SPU-Net achieved comparable results of sensitivity, DSC, mHD, and HD95. While comparable to U-Net, SPU-Net has the added benefit of providing uncertainty quantification in the form of an uncertainty map which is spatially aligned with the segmentation prediction. This uncertainty map closely matches clinical expectations, accurately reflecting areas where oncologists typically face difficulty in delineating meningiomas, such as regions overlapping with the skull, blood vessels, and the dural tail. These regions are known to have higher uncertainty in manual delineation, and SPU-Net’s results mirror this clinical challenge. By providing an interpretable outcome that highlights these uncertainties associated with segmentation, SPU-Net significantly enhances the potential for clinical adoption after future preclinical validation studies using a larger dataset, bridging the gap between automated systems and human expertise in the pursuit of precision medicine.

## Data Availability

The raw data supporting the conclusions of this article will be made available by the authors, without undue reservation.
